# Technical report on intra-operative trigeminal root mapping in percutaneous lesioning for trigeminal neuralgias

**DOI:** 10.1007/s00701-024-06323-4

**Published:** 2024-11-01

**Authors:** Marc Sindou, Andrei Brinzeu

**Affiliations:** 1 University of Lyon, 138 Avenue Lacassagne, 69003 Lyon, France; 2 University of Timisoara, Timisoara, Romania

**Keywords:** Trigeminal neuralgias, Radio, Frequency, Thermo, Rhizotomy, Percutaneous lesioning, Techniques, Intra, 0perative Neurophysiology, Trigemino, Facial reflexes, Blink, Reflex

## Abstract

**Purpose:**

Percutaneous lesioning-techniques for treating refractory Trigeminal Neuralgias not amenable to Micro-Vascular Decompression remain useful in neurosurgical practice. Success, avoidance of complications and reduction of side-effects depend on the accurate location of the lesion-maker especially for Radio-Frequency-Thermo-Rhizotomy (RF-Th-Rh).

Added to X-ray-guidance, Intra-Operative Neurophysiology can be of significant help to achieve optimal accuracy of the surgery.

Based on previous research, this article aims to describe the simplest way to use direct electrical stimulation of the trigeminal root to evoke clinically observable muscle responses allowing to precisely position the tip of the needle for accurate lesioning.

**Technique to evoke specific localizing muscle responses:**

Masticatory twitches can be easily produced by stimulating the motor root, through orthodromic conduction to the masticatory muscles.

Evoked Muscle Responses (EMRs) can be elicited in the facial nerve territory by stimulating the sensory rootlets, through Trigemino-Facial Reflexes’ pathways (TFRs). Responses in the Orbicularis Oculi is the well-known and readily used “Blink reflex”. On the contrary, TFRs in the lower territory of the facial nerve escaped clinical investigations not having been explored under direct stimulation of the trigeminal root. For both, stimulation at 5 c/s produces better observable twitches (because saccadic) than at 50 c/s which elicits tetanic contractions.

**Conclusion:**

The localizing-value of these facial EMRs (associated to evocation of paresthesias) and of the masticatory responses, justifies mapping the trigeminal root before lesioning. Their use could be extended to the other lesioning-techniques: not only Glycerol Neurolysis but also to Balloon Compression (to ascertain location of the trocar at the contact of the TGN inside the Meckel cave) and Open partial Rhizotomies (before deciding to cut the rootlets corresponding to the trigger-zone). This is of importance since lesioning-techniques are needed because not all trigeminal neuralgias are responsive to or even indications of Micro-Vascular Decompression.

## Introduction

Percutaneous lesioning-techniques for treating refractory Trigeminal Neuralgias (TNs) not amenable to or after failure of Micro-Vascular Decompression (MVD) are still useful in neurosurgical practice. Among them, Glycerol injection, Balloon compression, and Radiofrequency-Thermocoagulation are currently used. Success, avoidance of complications and reduction of side-effects depend essentially on the accurate location of the lesion-maker whatever the method performed. This is especially true for the Radio-Frequency Thermo-Rhizotomy (Rf-Th-Rh). In addition to the crucial use of intraoperative X-ray guidance, neurophysiology adds precision of the lesion in the location relative to the nerve fibers – thus improving accuracy and safety of the surgery. The present report aims to give the practical and simple way to evoke specific muscle responses with significant localizing value using direct stimulation of the trigeminal nerve (TGN) root. The article is a complement to the recent video-publication focused on the surgical procedure itself [2] its text gives the detailed neurophysiological protocol for trigeminal root mapping, as it was used in a previous publication but not precisely detailed [12].

## Methods

### Anatomical-Neurophysiological background

1. The safety of the Hartel’s percutaneous trajectory to reach the Foramen Ovale requires to precisely draw the corresponding cutaneous landmarks on the patient’s face [7].
2. Placement of the needle at level of the Meckel trigeminal cave needs rigorous X-ray control. [14]
3. Emission of cerebrospinal fluid may lack or—if present—is not specific of the correct needle placement in the trigeminal cave. Extremity of the needle may be too lateral in the crural subarachnoid space or too posterior in the cerebellopontine cistern [13].
4. Whereas targets are: the trigeminal cistern inside the trigeminal cave for Glycerol injection and the trigeminal ganglion of Gasser for Balloon compression, the target for RF-Th-Rh is the sensory rootlets located in the immediate retrogasserian portion of the trigeminal root—i.e., the so-called Triangular Plexus (TrPl) [13, 14]. Indeed, at the TrPl the trigeminal sensory fibers have a clearcut somatotopic organization [1, 5, 11].
5. The relative position within the TGN root, of the (uninsulated) tip of the needle/electrode can be identified intra-operatively by direct electrical stimulation of its constituting fibers.

***Direct stimulation of the motor root*** (i.e., the masticatory fibers) produces orthodromic motor responses in the masticatory muscles [3].

Further, ***direct stimulation of the sensory root fibers*** was found to elicit reflex motor responses in the masseter and the orbicularis oculi muscles [4]. Later in 1988 Kawamura et al. described, treating TNs with glycerol injection, the use of the blink-reflex to verify that the situation of the needle be adjacent to the trigeminal ganglion before injecting the glycerol into the trigeminal cistern [8].

In contrast with the well-demonstrated reflex in the upper facial nerve territory (the so-called blink-reflex) to our knowledge no reflex responses—at least to direct trigeminal root stimulation—were reported in the neurosurgical literature in the lower territory of the facial nerve: that is in Levator Labii and/or Orbicularis Oris. Their first report was in 1979 on the occasion of RF-Th-Rh surgery [13]*.* Demonstration of their Trigemino-Facial Reflex TFR nature was brought through intraoperative EMG-recordings and published in 1994 [12]. Because of the localizing-value of these (independently produced) facial Evoked Muscle Responses (EMRs): in Orbicularis, Levator Labii or Orbicularis Oris, attested by their respective topographic correspondence with the V1, V2 or V3 trigeminal root division(s), TFRs mapping was proposed for navigation within the TGN system. This proposal was based on a correlation study in a population of 441 consecutive patients operated on with the RF-Th-Rh procedure. The detailed description of the methodology used for that study and further refined through over 2000 other procedures since, is the object of this technical report. In these latter patients, with accumulated experience, responses in the form of TFRs were observed in more than three quarters of patients as opposed to the published series in which TFRs were observed in a little less than half the patients.

### Protocol for direct root electrical stimulation in the RF-Th-Rh procedure

The surgical technique followed was initiated by Professor William Sweet in Massachussets General Hospital at Harvard University in Boston and learned by the senior author (MS) during his fellowship in the late seventies [14]. Use of the Trigemino-facial reflexes, with observation of their clinical expression during surgery, was added to the procedure for helping navigation inside the TGN [10].

1. Because cannulation of the Foramen Ovale (FO) and thermal lesioning are painful, episodes of short-lasting general anesthesia, currently with Intravenous Propanidide* [Epontol; Laboratoires Théraplix, 46–52 Rue Albert, 75,640 Paris Cedex 13, France]* are needed. Level of anesthesia/analgesia should not impair corneal reflex examination. Noteworthy, local anesthesia would be counterproductive hindering the sensory testing.
2. Landmarks are measured and marked on the skin of the cheek as described by Hartel [7]. The tip of the needle should target – on the lateral X-ray view – the intersection between the clivus line and the upper petrous ridge, which corresponds to the Triangular Plexus (TrPl) projection.
3. Thereafter stimulation can begin, patient awakened. The stylet of the needle is replaced by the (unipolar) electrode, uninsulated over its distal 7mm. The reference needle-electrode (anode) is inserted subcutaneously in the middle of the hairline at the forehead as advocated by Sweet. Contact of the electrode tip with the sensory fibers is tested by checking the presence and topography of the paresthesias evoked by the root stimulation [14].

Stimulation at 5 Cycles/Sec (c/s) makes the triggered muscle responses more easily identifiable, than the classical one at 50c/s used by Sweet. This shift was decided in order to transform the provoked tetanic contractions (difficult to differentiate from mimic reactions to pain) into saccadic twitches (clinically easier to detect) [13]*.* For this a RFG5 Radionics RF-Generator, from Radionics Incorporation, 76 Cambridge Street, Burlington, Massachusetts 01803 USA was used; later this was taken up by Cosman and further by Boston Scientific*.*

(i) The *initial step* is to check the location of the electrode tip at level of the TrPl by looking at the masticatory responses to stimulation. Masticatory twitches appearing at low intensity (below 0.4 V) the electrode tip being at proximity of the Foramen Ovale (FO) on fluoroscopy, would warn for a too peripheral location. Conversely, masticatory twitches with the electrode tip beyond the upper petrous ridge would inform on a excessively deep location inside the cerebellopontine angle cistern.
(ii) The *following step* is to ascertain the appropriate location of the electrode, at level of the TrPl, in the sensory fibers corresponding to the trigger-zone. This is tested by checking, with the patient well awake the topography of the paresthesias evoked by the stimulation, also at a frequency of 5 c/s. Position is adjusted until paresthesias cover the painful area, optimally at an intensity below 0.4 V. Higher intensity would indicate electrode location far from the trigeminal rootlets.

Then, muscular twitches in the facial muscles as a response to the stimulation of the sensory fibers are carefully looked for.

The electrode position is adjusted until the paresthesias are felt covering the territory of the painful trigeminal division (V1, V2, and/or V3), and until the EMRs be located, respectively, in the Orbicularis Oculi, Levator Labii or Orbicularis Oris muscles, accordingly. One needs to bear in mind that EMRs are not always present.

Then, the thermo-lesion is performed by increasing step by step the temperature from 55° up to75° degrees for sequences of 60 s. The patient is awakened and tested between each lesion to verify the topography of the produced hypoesthesia. The corneal reflex is regularly checked with a fine cotonoïd pledget, and if the reflex diminishes, the procedure is halted.

Hypoesthesia in the skin and mucosae is tested by pinprick at each step. When the Trigger-zone is covered, preferably with some overextension, the procedure is considered completed.

As a side note, the senior author has observed that if the lesion is well performed the TFRs tend to disappear or are evoked at a higher intensity than at the begging of the surgery. This is considered to be prognostic element for a good result.

## Empirical evidence supporting the trigeminal root mapping methodology

This methodology previously tested in a controlled setting and already published in 1994 [12] has been applied since then in over 2000 patients with similar results. The empirical data supporting these recommendations can be summarized as follows:

1. Triggered twitches in masticatory muscles at low intensity (below 0.4 V) were observed in all the procedures on or soon after crossing the Foramen Ovale, testifying of the (too) peripheral situation of the electrode tip. Triggered masticatory twitches, also at low intensity, were provoked each time the electrode tip was 5mm beyond the upper petrous ridge, testifying of its (too) deep situation in the cerebellopontine cistern, in close vicinity to the pars minor (= motor fibers) of the trigeminal root.
2. Of the 459 procedures performed, clear-cut EMRs were clinically-observed in 203 (i.e., 44.2%). Comparing features between the group with EMR + and the one with EMR -, the threshold of stimulation to evoke paresthesias, as well as the duration of the thermo-lesioning for obtaining a marked hypoesthesia, were significantly lower in the EMR + group (p = 0.01 for both). This testifies of a closer situation of the electrode tip to the sensory root when EMR is present.

Concerning the localizing-value of the EMRs in regard to the sensory rootlets, there was a good correlation between the location of the facial EMRs and the targeted division(s) of the trigeminal root. Correlation was positive between the EMRs (in the Orbicularis Oculi, the Levator Labii, or the Orbicularis Oris) and – respectively—the (V1, V2, V3) topography of the concerned trigeminal territory, in 95% of the procedures for evoked paresthesias and in 96% for the hypoesthesia resulting from the thermo-lesioning.

## Discussion

**I. Practical experience** shows that intra-operative mapping of the trigeminal root not only for eliciting (direct) masticatory twitches but also (reflex) evoked muscle responses in the facial nerve territory can be integrated to the classical RF-Th-Rh procedure for making it the more selective as possible.

Twitches in the masticatory muscles under stimulation at 5c/s are always easily recognizable. Regarding facial EMRs the easiest ones to observe are the responses in the Orbicularis Oculi (so-called Blink-reflex); EMRs in the Levator Labii and Orbicularis Oris were more difficult to detect.

During the study these different EMRs manifested independently depending on the location of the uninsulated tip of the electrode, respectively in the V1, V2 and V3 components of the triangular plexus where somatotopy is known to be optimal [1]. Noteworthy, evocation of twitches in the Levator Labii muscle was sometimes difficult (in approximately one -third of the patients) to get without contamination to some degree by twitches in the Orbicularis Oculi superiorly and Orbicularis Oris inferiorly. However, the localizing value of these various EMRs was corroborated by a good topographical concordance (in 95% of the cases) with the projection of the paresthesias felt by the (awake) patient.

In the formalized related study [12], EMRs could be observed in only 44.2% of the procedures. Absence can be retrospectively interpretated due to situation of the uninsulated tip of the electrode outside the targeted trigeminal rootlets, in spite of X-ray landmarks considered appropriate.

Noteworthy, these EMRs in the facial nerve territory were always easy to differentiate from the twitches in the masticatory muscles, those latter being linked to the direct stimulation of the efferent fibers of the trigeminal motor masticatory root, and their orthodromic conduction.

Both of these neurophysiological manifestations: those in the masticatory muscles and those in the facial muscles, can be useful for navigation and guidance inside the TGN system.

1. *Twitches in the masticatory muscles* at low intensity mean a situation of the electrode too close to the motor root fibers. Masticatory twitches—the electrode being in the vicinity of the Foramen Ovale—would indicate an inappropriate, peripheral location of the electrode. Masticatory twitches—the electrode tip beyond the Upper petrous ridge/Clivus line on lateral X-ray -would warn on a too deep penetration inside the cerebellopontine angle, in the vicinity of the trochlear nerve.
2. Due to their localizing-value, *facial EMRs* elicited by root stimulation at low intensity do constitute an objective check of the appropriate location of the electrode in the sensory fibers of V1 for EMRs in Orbicularis Oculi, V2 for EMRs in Levator Labii, and V3 for EMRs in Orbicularis Oris. Importantly, an intense blink-reflex response at low intensity of stimulation should warn of the proximity of V1 fibers, and so of a high risk of corneal anesthesia after thermo-lesion.

**II. Main limitation of the study** was that the muscles’ responses to direct root stimulation were only identified on mere clinical observation. Obvious clinically-observable EMRs were obtained in only half of the cases. In a personal study (limited to 15 patients) in which EMG-recordings were performed to demonstrate and characterize the electrophysiological events, EMRs were present in 11 of the 15 explored cases [12]. In this study with calculation of the latencies of the electrophysiological events, the EMG-responses—including those in the lower facial muscles—were found coherent—with brainstem Trigemino-Facal Reflexes, and their localizing value electro-physiologically demonstrated.

In spite of these limitations, the attempt in this article to establish neurophysiological criteria possessing localizing-value provides a supplementary mean – objective in nature because clinically-observable—to make the RF-Th-Rh procedure more selective.

### III. Use of TFR mapping in other trigeminal lesioning-procedures

In the neurosurgical domain, according to literature review, it should be recalled that Kawamura et al. reported, as soon as 1988, the use of the blink-reflex to intracranial stimulation of the TGN for improving accuracy of the method of *Neurolysis with Glycerol injection in the trigeminal cistern* [6]. Purpose of the authors was to verify the location of the needle extremity close enough to the trigeminal ganglion before injecting the glycerol into the trigeminal cistern [8]. This wise precaution was also to decrease the risk of glycerol diffusion in the neighboring subarachnoid spaces.

Same protocol can be proposed for securing the *Balloon compression technique* [9], specifically placement of the balloon inside the dural lodge of the Meckel cave in contact with the contained trigeminal ganglion. Stimulation of the TGN with obtention of a Blink-reflex should be obtained by inserting a stimulation probe into the trocar prior to the introduction of the Fogarty catheter. Of course, the classical X-ray control on lateral view, with the balloon inflated with 0.5–1 ml of contrast- medium showing a piriform shape, remains the most robust testimony of its good location inside the trigeminal cave and posteriorly at the trigeminal porus.

The same neurophysiological principle could be applied for *open partial rhizotomies,* in order to identify by direct stimulation of the exposed root—under the surgical microscope and using EMG-recordings—the topographical systematization of its constituting rootlets before deciding of their selective interruption.

## Conclusion

In spite of some limitation in reliability, trigeminal root mapping was found of ***practical usefulness*** as an objective marker of the location of the electrode within the TGN system. Trigeminal root mapping is easy to perform without any additional technical constraint. Added to the rigorous protocol of the procedure as developed by Sweet in the seventies, this neurophysiological mapping can be helpful, especially in unconscious or drowsy and/or confused patients.

## Data Availability

No datasets were generated or analysed during the current study.
